# Nerve Transfers for Brachial Plexus Reconstruction in Patients over 60 Years

**DOI:** 10.3390/jpm13040659

**Published:** 2023-04-12

**Authors:** Andreas Gohritz, Gregor Laengle, Anna Boesendorfer, Bernhard Gesslbauer, Clemens Gstoettner, Olga Politikou, Agnes Sturma, Oskar C. Aszmann

**Affiliations:** 1 Clinical Laboratory of Bionic Extremity Reconstruction, Department of Plastic, Reconstructive and Aesthetic Surgery, Medical University of Vienna, Währinger Gürtel 18-20, 1090 Vienna, Austria; 2 Department of Plastic, Reconstructive and Aesthetic Surgery, Hand Surgery, University Hospital, Petersgraben 4/Spitalstrasse 21, 4031 Basel, Switzerland; 3 Bachelor’s Degree Program Physiotherapy, University of Applied Sciences FH Campus Wien, Favoritenstrasse 226, 1100 Vienna, Austria

**Keywords:** nerve reconstruction, elderly patients, brachial plexus injury, nerve transfer, postoperative training, EMG-feedback

## Abstract

Negative expectations regarding nerve reconstruction in the elderly prevail in the literature, but little is known about the effectiveness of nerve transfers in patients with brachial plexus injuries aged over 60 years. We present a series of five patients (1 female, 4 male) aged between 60 and 81 years (median 62.0 years) who underwent nerve reconstruction using multiple nerve transfers in brachial plexopathies. The etiology of brachial plexus injury was trauma (*n* = 2), or iatrogenic, secondary to spinal surgical laminectomy, tumor excision and radiation for breast cancer (*n* = 3). All but one patient underwent a one-stage reconstruction including neurolysis and extra-anatomical nerve transfer alone (*n* = 2) or combined with anatomical reconstruction by sural nerve grafts (*n* = 2). One patient underwent a two-stage reconstruction, which involved a first stage anatomical brachial plexus reconstruction followed by a second stage nerve transfer. Neurotizations were performed as double (*n* = 3), triple (*n* = 1) or quadruple (*n* = 1) nerve or fascicular transfers. Overall, at least one year postoperatively, successful results, characterized by a muscle strength of M3 or more, were restored in all cases, two patients even achieving M4 grading in the elbow flexion. This patient series challenges the widely held dogma that brachial plexus reconstruction in older patients will produce poor outcomes. Distal nerve transfers are advantageous as they shorten the reinnervation distance. Healthy, more elderly patients should be judiciously offered the whole spectrum of reconstructive methods and postoperative rehabilitation concepts to regain useful arm and hand function and thus preserve independence after a traumatic or nontraumatic brachial plexus injury.

## 1. Introduction

Traumatic brachial plexus injuries mostly affect active male individuals in their second or third decade of life and cause enormous morbidity. Nerve transfers have been established as standard treatment, in addition to microsurgical neurolysis, direct repair and interposition grafting [1]. Highly positive outcomes have been reported in numerous cohorts, such as 96% of individuals who had undergone nerve transfers, who attained at least M3 elbow flexion or greater, and 74% whose postoperative shoulder abduction was at least M4 or greater [2], with similar success rates for various other surgical techniques for nerve reconstruction [3,4,5]. However, negative attitudes prevail in the literature, correlating advanced age with inferior results after reconstructive operations of the brachial plexus, despite a paucity of clinical data on this specific subgroup [6,7,8,9,10].

Responsible factors for nerve regeneration have been studied intensively in animal models, with clear evidence of age-related impairment in nerve regeneration [11]. Functional testing in elderly subjects displays decline in tetanic force, thermal and tactile sensation, and electrophysiological parameters. This correlates with an altered morphology of axonal structures in elderly individuals. Histological analysis confirms a reduced number and density of axons and of their cytoskeletal elements, and thinner myelin sheath of regenerated nerves with progressing age. Age-related difference in function and morphology may involve the nerve and its target organ, but also systemic factors of aging. Kovačič et al. believe that one major reason for slowing of nerve regeneration in aged individuals is an alteration in neural pathways responsible for guidance of the sprouting axons [11]. Specifically, Schwann cells display a reduced reactivity for promoting nerve regeneration after nerve injury. The intrinsic potential of axonal growth seems to be preserved. Moreover, changes in the target organ itself (e.g., muscle) lead to impaired neurotrophic signaling for support of axonal structure and function.

Although there are limiting factors for nerve regeneration in senescence, there may be a role for nerve reconstruction procedures in improving functional outcome. As a general rule, nerve transfers are a reliable method in brachial plexus surgery with specific advantages over nerve grafting [3]. A shorter time to reinnervation can be achieved due to the closer proximity to the target muscle and the single coaptation site, without necessity of a nerve graft. Fast muscle reinnervation might thus compensate for the impaired regenerative potential in older patients with brachial plexus injuries. Recent histomorphometry studies by our group revealed that motor fibers, surprisingly, only account for about 10% of the overall axon count, even in “pure” motor nerves [12]. This may emphasize the probably underrecognized importance of sensory afferences for motion control, which should be targeted by specific sensory-cognitive rehabilitation, especially in elderly patients.

## 2. Objective

The purpose of this study was to investigate whether elderly patients with brachial plexus injury, defined as aged over 60 years, are suitable candidates for surgical restoration of upper extremity function using nerve transfers.

## 3. Patients and Methods

In this retrospective, single-center analysis, all patients who met the following criteria were included: (1) received nerve reconstruction by the senior author using nerve transfers for a brachial plexus injury within the last decade; (2) aged 60 years or above; and (3) were available to long-term (>1 year) follow-up. This study has been approved by the institutional review board and all patients gave their informed consent.

All patients underwent postoperative physiotherapy and occupational therapy following a structured rehabilitation program [13]. An emphasis was put on early mobilization, improvement of cortical representation of the affected arm and electromyography-feedback training. This enabled the patients to reformat their individual motor matrix and thus learn how to activate the new nerve connections as soon as possible. Task-oriented training was usually introduced parallel to strengthening exercises and was designed towards integrating the new arm and hand function into daily life activities.

Functional outcome of the reinnervated muscle group was assessed with the Medical Research Council grading system and measurement of active joint mobility. According to the distribution, normality data is displayed as either mean ± standard deviation (SD) or median ± interquartile range (IQR) for descriptive analysis. For each strength level (M0–M5), the range of motion of the respective joint is indicated.

## 4. Results

A group of five patients (one female, four male) aged between 60 and 81 years (median 62.0 years, IQR 61.0–71.5) was identified. Specific patient characteristics are displayed in Table 1. The causes of brachial plexus injuries included trauma, such as bike or motorbike accidents (*n* = 2) and iatrogenic lesions through spinal surgical laminectomy, tumor excision and radiation for breast cancer (*n* = 3).

**Figure 1:**
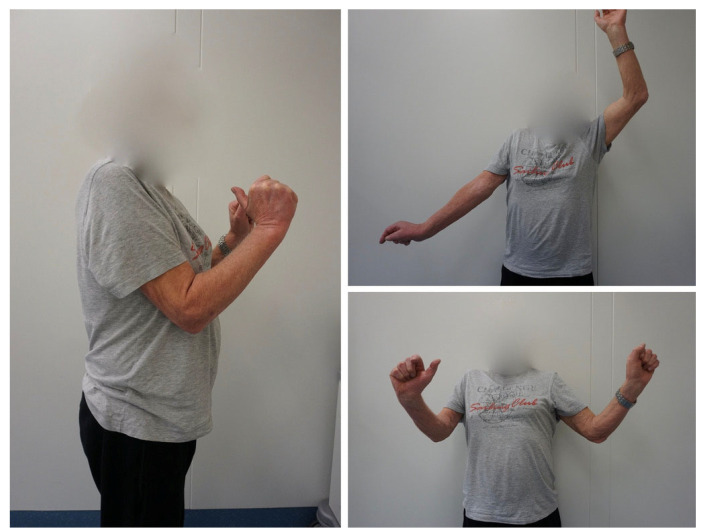
Postoperative result of a triple nerve transfer (spinal accessory nerve to suprascapular nerve, radial nerve fascicle to axillary nerve, median nerve fascicle to brachialis muscle) in an aged patient after complete paralysis of his deltoid, supra-/infraspinatus, biceps and brachialis muscle, showing good shoulder abduction and elbow flexion at M3 strength level.

All but one patient underwent a one-stage reconstruction including neurolysis and extra-anatomical nerve transfer alone (*n* = 2) or combined with anatomical reconstruction using sural nerve grafts (*n* = 2). One patient had anatomical brachial plexus reconstruction first and second stage nerve transfer. Nerve transfers were performed as single (*n* = 2), double (*n* = 1), triple (*n* = 1) or quadruple (*n* = 1) nerve or fascicular transfers. Mean delay of surgery after injury was 13 months (range 0–31). It is noted that one patient had a progressive brachial plexus palsy after irradiation therapy (P3) and another patient had immediate reconstruction post tumor resection in a one-stage procedure (P5). Detailed description of surgical procedures and outcome is shown in Table 1. First reinnervation signs assessed with surface electromyography occurred within 6 months in all patients. Overall, successful outcomes, defined as a restored muscle strength of at least M3 of the addressed muscles, was achieved in all cases within a minimum follow-up of 12 months (median 23 months, IQR 18–81). The best achievable outcome was grade M3 (range of motion 40–90°) for shoulder abduction and tended to be better with elbow flexion up to level M4 (range of motion 90–130°, Figure 1).

Complications included transient painful paresthesia in the hand (*n* = 1), moderate weakness of index finger flexion improving over time (*n* = 1) and persistent co-contraction of the brachialis muscle and wrist/finger flexors (*n* = 1).

## 5. Discussion

This retrospective study reports on a cohort with brachial plexus injuries in patients aged over 60 years in whom arm and hand function was principally restored through multiple nerve transfers. The proportion of brachial plexus patients in this elderly age group is small. Epidemiological studies show that brachial plexus injuries mostly strike young active males due to road-traffic accidents, but little is reported on elderly individuals. In a systematic review and meta-analysis of 3032 individuals from 10 studies conducted in 8 countries, 93% were male and pooled age was 29 years (range 24–34), but no detailed data on elderly patients was presented [14]. Jain et al. reported on 304 surgically treated patients in India, including only four (1.3%) aged over 60 years, again without specific information on this elderly subgroup [15].

Although our group size is limited, the patient characteristics suggest that etiology of injury in elderly patients differs from younger brachial plexus patients. While they can also suffer from traumatic accidents, reflecting their high activity level in sports and leisure time, a considerable proportion, three out of five in our cohort, were paralyzed due to age-typical non-traumatic reasons, i.e., malignancy and associated medical treatments (e.g., radiation) or other sequelae of age-related pathologies. However, supporting hand function through minor neurosurgical procedures, such as nerve transfers, can help patients maintain an active and healthy lifestyle even in their “aged” years.

A number of experimental studies [11,16] and clinical series have correlated increased age with decreased functional outcome, setting the threshold for “young” versus “old” at age levels around 35–40 years [6,8,9]. In a review of 194 patients who had undergone reconstruction of their musculocutaneous nerve injuries, those who were aged younger than 20 years revealed significantly stronger elbow flexion strength than individuals aged 40 years or older [9]. The analysis of 146 axillary nerve repairs (maximum age 72 years) demonstrated a significant decrease in successful results with increasing patient age, while 83% of individuals aged younger than 20 years achieved M4 power of shoulder abduction by deltoid muscle power, whereas the success rate was only 63% in those older than 35 and 61% aged over 40, respectively [6]. Likewise, in a cohort of 33 patients after axillary nerve reconstruction, 8 out of 14 aged younger than 25 years had favorable results in contrast to only 8 out of 19 who were 25 years or older [7].

Similar results were also documented for individuals of advanced age after nerve transfer operations. Weaker biceps strength after using a single ulnar fascicle transfer in patients with brachial plexus injuries led to the development of a double fascicle transfer by adding a median nerve fascicle to brachialis transfer [17,18]. Consecutively, all patients achieved elbow flexion of at least M4 (age range 17–61 years). Lee et al. revealed in their study that deltoid muscle power restored by triceps-to-axillary neurotization decreased with increase in patient age (range, 16–79 years) [10]. While 11 out of 12 individuals who were 39 years or younger regained at least antigravity muscle strength, similar results were only achieved by five out of nine patients who were 40 years or older. Four of the five results with only M2 or less muscle strength occurred in patients aged over 50, and none of the patients beyond 50 years of age achieved a muscle strength of M3 or greater.

On the other hand, more recently, three cases of successful nerve transfers of complex nerve lesions in elderly patients were published, of whom two were septuagenarians. Willis and Ahmadi in 2019 reported on a patient aged late 60s with radial-to-axillary nerve transfer, resolving axillary nerve palsy due to proximal humerus fracture-dislocation treated with hemiarthroplasty [19]. Jiang and Lio in 2016 reported on successfully restoring M3 elbow flexion by a phrenic nerve transfer in the treatment of a patient aged early 70s with brachial plexus avulsion injury due to traffic accident [20]. Johnson and Wolfe documented the long-term follow-up of a patient aged mid 70 s who underwent triple nerve transfer (triceps to axillary, spinal accessory to suprascapular and ulnar to musculocutaneous nerve) 16 weeks post brachial plexus injury due to a skiing accident. At the final follow-up, shoulder abduction measured 65° with M4 muscle strength though limited by arthritis of the glenohumeral joint. Biceps and brachialis muscle strength for elbow flexion were both M5, and good muscle reinnervation characterized by motor unit recruitment of the previously paralyzed muscles was proven by electro-diagnostics [21].

These results are comparable with the favorable outcomes in our small series of five patients, which showed a high rate of successful restoration of at least antigravity muscle power for shoulder abduction and elbow flexion after nerve transfers. We attribute our positive results to a relatively short delay between nerve injury and muscle reinnervation, which is acknowledged as a crucial factor for functional recovery after nerve repair. Although increased patient age has been determined as an important negative factor in scientific studies with regard to impaired axonal regeneration, cortical reorganization and cognitive adaption, nerve transfers offer the advantages of shortened reinnervation distance and optimal donor nerve health [22,23]. The coaptation site close to the target muscle might also be an important factor for a powerful reinnervation, since a defined motor axon group can be shifted to a muscular branch without the risk of unintended motor axon loss into a sensory nerve branch. In our series, four of five patients were male, which reflects the gender disbalance of brachial plexus injuries, which mainly affect men [15]. Even though the literature suggests a pro-regenerative effect of androgen hormones in nerve regeneration, there is no clear evidence for superior results in males [24,25,26]. For evaluation of a clinical gender difference in peripheral nerve regeneration a bigger cohort with a representative number of female patient would be necessary.

**Figure 2:**
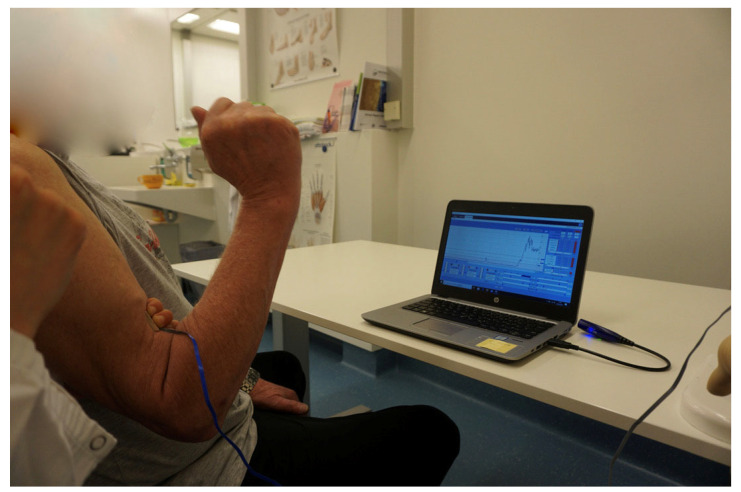
EMG-feedback training is a fundamental part in neuromuscular rehabilitation after nerve transfers, especially in older patients. The visualization of muscular activity facilitates relearning of muscular control.

Furthermore, innovative rehabilitation concepts, such as early structured motor training using surface electromyography biofeedback (Figure 2), have demonstrated an ability to facilitate and fasten the cognitive adaption and relearning following nerve transfer procedures after brachial plexus injuries, making them a promising treatment method for otherwise healthy individuals who have sustained a lesion of their brachial plexus, in order to enable faster recovery of key upper extremity functions [13,27,28]. Compared to younger individuals, post-surgical rehabilitation is more demanding for older patients and hence requires higher frequency training. Nerve regeneration may be diminished in advanced age, yet reconstruction can nevertheless markedly improve arm and hand function. In particular, nerve transfers appear advantageous for healthy, older patients with traumatic brachial plexus palsy by enabling faster muscle recovery.

The coordination of movements, which tends to be worse in the older population, was not specifically analyzed in this article [29]. Marchini et al. conducted a study to investigate differences in motor output variability during tasks involving the simultaneous dorsiflexion of both feet between a group of young (average age of 28 years) and old (average age of 67 years) participants. They showed a significantly better motor coordination of the young adult group compared to the old adult group. The performance of long-term training (>1 year) did not have a significant effect on the results of the older participants. This may display the limitations of cognitive motor rehabilitation in senescence. There is no general recommendation for a specific age limit to achieve favorable outcome in peripheral nerve surgery. Since biologic age differs inter-individually, the decision to perform nerve transfers in patients over 60 should be made on a personalized basis.

It is remarkable that active learning of new motor skills and training brain plasticity through activities like dancing have been shown to improve physical and cognitive function and slow the decline in quality of life in geriatric individuals with dementia and Alzheimer’s disease [30]. Thus, even in people of advanced age, combined neurocognitive training after nerve transfer surgery can help maintain an active and autonomous lifestyle. Accordingly, in a blinded randomized controlled study, 40 patients with a mean age of over 61 years, who had been diagnosed with Parkinson’s disease, significantly profited from a specialized sensory-motor training, regarding improved sensory and motor function of their hands and upper extremities at levels 1 to 3 of the Hoehn and Yahr Scale [31].

Elderly patients may soon become the most relevant population to benefit from cognitive nerve transfers to treat spasticity or paresis in upper motor neuron syndrome, i.e., due to stroke [32,33]. As life expectancy continues to increase world-wide, hand and peripheral nerve surgeons will be confronted with increasing numbers of specific upper extremity problems in geriatric patients [34]. A multi-center study may expound upon the benefits of complex nerve reconstruction, including transfers, in this growing subgroup of patients.

## Figures and Tables

**Table 1:** Patient characteristics and outcome.

Patient	Age (y), Gender	Diagnosis	Aetiology	Operations (Delay (Months))	Follow-up (Months)	Outcome (Range of Motion)
P1	61, m	Extended upper BPI: - C5/6 infraganglionary lesion - C7/8 supraganglionary avulsion - Palsy of supra-/infraspinatus, deltoid, biceps, triceps, pectoralis major and trapezius	Motorbike accident	1. Operation (3 m): Sural nerve grafting: - C5 to suprascapular nerve and superior trunk/posterior division/axillary nerve (2 × 7 cm) - C6 to superior trunk/anterior and posterior division (4 × 5 cm) 2. Operation (13 m): Nerve transfer: - Median nerve fascicle to brachialis muscle	19	Shoulder abduction M3 (30°) Elbow flexion M3 (90°) Elbow extension M2 (0°) Persistent co-contraction of the brachialis muscle and wrist/finger flexors
P2	81, m	Upper BPI: - C5/C6 lesion - Palsy of supra-/infraspinatus, deltoid, biceps, brachialis	Iatrogenic lesion due to bilateral hemi-laminectomy C4/5 and foraminotomy C4–6 for spinal stenosis	1. Operation (16 m): Triple NT: - Spinal accessory to suprascapular nerve - Radial nerve triceps branch to axillary nerve - Median nerve fascicle to brachialis muscle	17	Shoulder abduction M3 (40°) Elbow flexion M3 (110°)
P3	62, f	Upper BPI: - Superior trunk enlargement - Palsy of biceps brachii	Radiotherapy (50 Gy), chemotherapy due to breast carcinoma	1. Operation (31 m): Infraclavicular neurolysis Nerve transfer: - Ulnar nerve fascicle (FCU) to both biceps muscle branches	23	Shoulder abduction M3 (20°) Elbow flexion M4 (100°)
P4	62, m	Upper BPI: - C5/6, superior trunk and phrenic nerve lesion - C5/6 non-reconstructable - Palsy of supra-/infraspinatus, deltoid, biceps, brachialis, diaphragm (one-sided)	Bike accident, fractures of cervical and thoracic spine	1. Operation (5 m): Multiple nerve transfers: - Ulnar nerve fascicle (FCU) to biceps muscle - Median nerve fascicle (FCR) to brachialis muscle - Radial nerve triceps branch to axillary nerve - Spinal accessory nerve to suprascapular nerve - Dorsalis scapulae nerve to axillary nerve (ETS) - Phrenic nerve to C7 root (ETS)	126	Shoulder abduction M3 (90°) Elbow flexion M4 (130°) Painful tingling/paresthesia in the hand
P5	61, m	Tumor (Fibromatosis) upper trunk hypesthesia in the arm/hand, no motor deficits	Surgical resection	1. Operation (0 m): Tumor resection including root C5/C6 and phrenic nerve Nerve grafting: - C5 to upper trunk and suprascapular nerve (MACN) Nerve transfers: - Median nerve fascicle (FCR) to biceps muscle - Ulnar nerve fascicle (FCU) to brachialis muscle	36	Shoulder abduction M2 (20°) Elbow flexion M3 (100°) Reduced flexion of the index finger

Abbreviations: f: female, m: male, BPI: Brachial plexus injury, ETS: End-to-side, FCU: Flexor carpi ulnaris, FCR: Flexor carpi radialis, MACN: Medial antebrachial cutaneous nerve.

## Data Availability

Data is contained within the article.
